# Digital bioethics: exploring an emerging field

**DOI:** 10.1007/s11019-026-10347-1

**Published:** 2026-04-16

**Authors:** Georg Starke, Wolf-Tilo Balke, Lasse Benzinger, Oliver Buchholz, Manuel Burghardt, Lukas J. Meier, Emilian Mihailov, Eva Seidlmayer, Robert Ranisch, Frank Ursin, Effy Vayena, Sabine Salloch

**Affiliations:** 1 Institute of History and Ethics in Medicine, Technical University of Munich, Ismaninger Str. 22, 81675 Munich, Germany; 2 Institute for Information Systems, Technical University of Braunschweig, Braunschweig, Germany; 3 Institute for Ethics, History and Philosophy of Medicine, Hannover Medical School, Hannover, Germany; 4 Health Ethics and Policy Lab, ETH Zurich, Zurich, Switzerland; 5 Institute of Computer Science, Leipzig University, Leipzig, Germany; 6 Edmond & Lily Safra Center for Ethics, Harvard University, Cambridge, USA; 7 Research Centre in Applied Ethics, Faculty of Philosophy, University of Bucharest, Bucharest, Romania; 8 ZB MED–Information Centre for Life Sciences, Cologne, Germany; 9 Faculty of Health Sciences, University of Potsdam, Potsdam, Germany

**Keywords:** Bioethics, Methodology, Advanced data science, Experimental philosophical bioethics, Digital Humanities

## Abstract

The uptake of social science methods by bioethics significantly expanded its methodological spectrum, raising new theoretical, methodological, and practical questions. Recently, we are witnessing another trend, adding advanced data science methods to bioethics’ toolkit to aid, for example, in online data analysis, support scholarly writing, and inform clinical ethics. This article explores the emerging field of Digital Bioethics across its dimensions by analysing the tangled relationship between topics and methods, highlighting intersections between Digital Bioethics and Bioethics of the Digital, and advocating for a methods-based definition of the field. The use of advanced data science methods within bioethics must be interpreted in the context of the use of Artificial Intelligence (AI) in health care. At the same time, it presents unique opportunities and challenges. Defining, and thus demarcating, Digital Bioethics can create support for the new field but also requires navigating trade-offs. To do so, we take four kindred academic fields as points of comparison (Digital Humanities, Experimental Philosophical Bioethics, computational medicine and digitised biology) to analyse what each of them teaches for critically assessing and further developing Digital Bioethics. The article discusses potential pitfalls and concludes with recommendations on how the field can fully develop its potential to promote bioethical research and argument. Furthermore, the article discusses how a critical reflection of the use of AI methods within bioethics itself will also contribute to the ethical oversight of increasingly AI-driven branches of healthcare.

## Bioethics adopting digital methods

The history of bioethics as we know it today is usually described as a response to significant advances in the biomedical sciences (e.g., in transplant, intensive, or reproductive care) and societal developments that led to the emergent need for ethical guidance, expertise, and counselling (Jonsen 2003). In its early years, the discipline was largely dominated by scholars from theology and philosophy as well as by legal experts and physicians who met in early commissions that laid the foundation for the more differentiated structures of ethical oversight established later. As is well-known, the methodological spectrum of bioethics was significantly expanded in subsequent decades by the introduction of methods from the empirical social sciences (Borry et al. 2005) – a process that was accompanied by extensive and fruitful debates on the interaction between descriptive and normative elements in applied ethics (Leget et al. 2009; Molewijk et al. 2004). Debates on proper methods and standards for research appropriately considering the interdisciplinary nature of bioethics work have considerably progressed over the years leading to a broad array of approaches, guidelines, and training programs in Empirical Bioethics research (Wangmo et al. 2024; Ives et al. 2018).

In recent years, the rapid advancement of computational and data science methods has fuelled methodological progress across numerous research branches. Within medicine, basic science (e.g., experiments with cells, animals and other models, or omics research) as well as research involving human participants have significantly profited from advances in computer science, machine learning, and bioinformatics. In the clinical context, data-driven support systems are increasingly integrated into various medical disciplines accompanied by the introduction of applications that might be used by patients themselves for preventive, diagnostic, therapeutic and other purposes. In addition, public discourses about medical treatments, technologies and the practice of healthcare are increasingly held in digital arenas such as online forums and social media.

The AI-driven transformation of healthcare and biomedical research (often characterised as being “disruptive”) does not leave the bioethics community untouched, and ethical reflection on the digital transformation of healthcare has become an increasingly prominent topic in bioethics.^1^ Medicine and healthcare are becoming progressively “infocentric”, raising questions about what defines a human being as a person (Vică 2018). At the same time, the field also increasingly embraces advanced digital methods in its own methodological repertoire. During the last few years, a growing number of bioethics publications reported results from studies that operate with advanced data science methods or discuss the employment of such methods for various ethical tasks. To name just a few examples, Schneider et al. used methods such as online sentiment analyses or automated topic ranking to explore bioethical issues, such as the use of CRISPR-Cas9, in the context of social media. They also present an account of Digital Bioethics that draws on methods from the (digital) social sciences, arguing that such research aligns with the established aims of Empirical Bioethics (Schneider et al. 2023). Spitale et al. referred to committee protocols related to the Dutch termination of life act to train AI models for classifying cases and for fine-tuning an LLM for generating new, fictional scenarios (Spitale et al. 2023). Within clinical ethics, a proof-of-concept study developed a Machine Learning Model that operationalises the principles of autonomy, beneficence and non-maleficence for ethically challenging clinical cases (Meier et al. 2022). Other intensely debated topics from clinical ethics refer to the automated prediction of incapacitated patients’ preferences (Earp et al. 2024; Earp et al. 2025; Benzinger et al. 2024), the use of Large Language Models (LLM) to support patient information (Allen et al. 2024; Mirza et al. 2024) or demonstrating consent for biobanking with blockchain and generative AI (Barnes et al. 2024). For bioethics’ academic practice, finally, first steps have been taken to fine-tune LLMs for text production in ethical analyses (Porsdam Mann et al. 2023), raising questions not only for research ethics and transparent reporting but also with view to the models’ ingrained stances towards morality (Porsdam Mann et al. 2024; Pfeffer et al. 2025; Jiao et al. 2025).

Already today, digital approaches in bioethics exhibit a high degree of heterogeneity, with studies differing substantially in their methods, objectives (e.g., research, consultation, clinical practice, education), and stages of development. First discussions of concepts such as Digital Bioethics (Schneider et al. 2023), Big Bioethics (Pacyna and Sharp 2022) or Computational Bioethics (Rahimzadeh et al. 2022) highlight the growing interest in this emerging field. Given the rapid advancements in medical AI and AI-driven research, digital approaches to bioethics are likely to gain further traction in the next years. However, a joint definition and a systematic analysis of theoretical foundations, such as the relation between digital methods and topics, remains lacking.

Against this background, our article aims to programmatically explore the nuances associated with the concept of “Digital Bioethics”. By this term, we refer to *an interdisciplinary field addressing bioethical questions in light of a normative background using methods enabled by advanced digital technologies*. Depending on the application, these methods may function either as extensions of established empirical bioethics methodologies or as components of novel methodological approaches that introduce new epistemic assumptions. “Normative background”, in our definition, refers to ethical theories, concepts or arguments that serve for the evaluation of a concrete normative question. In its basic tenets, our tentative definition is in line with previous analyses of the field, as for instance provided by Schneider et al., who highlight that established empirical methods have acquired digital formats and that automated approaches for the analysis of massive amounts of data can be made fruitful for the bioethics domain to trace how bioethical issues take shape online (Schneider et al. 2023). Salloch and Ursin employ the term “Digital Bioethics” for “all attempts making use of advanced data technology for addressing bioethical research questions” (2023). Their understanding is insofar narrower than the definition proposed in this article as they refer to “bioethical research questions” instead of “bioethical questions”. The latter might also include more practice-oriented tasks, e.g., in ethics guidance and consultation. On the other hand, Salloch and Ursin do not so much include a focus on bioethics’ normative dimension and issues of normative-empirical interaction in Digital Bioethics.

As simple as our definition might sound, numerous questions arise when attempting to demarcate and characterise the field. As the result of an interdisciplinary consensus process involving scholars from diverse disciplines (e.g., bioethics, philosophy, informatics and data science, Digital Humanities), this article outlines and examines key challenges and decisions necessary to further shape and conceptualise the emergent field of Digital Bioethics. In addition, we draw on insights from other scientific branches to discuss opportunities and pitfalls of adopting data science methods. Finally, the article addresses critical factors required for Digital Bioethics to further flourish as an academic subfield. We believe that such flourishing constitutes an important scientific desideratum for bioethics. Only by enlarging the field’s methodological toolkit can we ensure that important digital information does not remain untapped, allowing scholars to address novel digital phenomena as well as old ethical questions with innovative answers.

## Drawing the line: advantages and disadvantages of defining digital bioethics

Undisputedly, there is methodological continuity between Empirical Bioethics and Digital Bioethics (Schneider et al. 2023; De Proost and Provoost, 2024), especially if we consider instances where collection and analysis of qualitative and quantitative data can be supported by advanced data technology. However, this continuity should not obscure important differences at the level of methodology. Digital Bioethics does not merely introduce new tools for collecting or analysing empirical data. Rather, some digitally enabled approaches alter the epistemic conditions under which bioethical knowledge is produced. These include research at scales that preclude interpretive exhaustiveness, in light of massive data sources, for instance from scraped social media, that have so far not been workable in research without the use of Machine Learning and related technologies.^2^ Novel technological tools further enable automated analytical processes that may shape human judgement and academic writing on ethical analyses, as well as the computational representation or simulation of moral reasoning itself (Schacht and Lanquillon 2025). In such cases, digital methods do not simply enhance existing empirical methodologies but give rise to new methodological configurations that warrant explicit reflection. Questions of scale and automation but also, for example, of simulation and immersion in virtual experiments, constitute valid reasons to explore Digital Bioethics as a distinct field of study. In addition, novel forms of stakeholder participation and of interdisciplinary cooperation, between ethics and social sciences or between ethics and informatics, might prompt us to reflect on differences between (conventional) Empirical Bioethics and Digital Bioethics.

Against the background of such differences, we see added value in defining Digital Bioethics as a specific field of study. A tentative definition therefore might serve as a starting point for critical debates on the prospects of using advanced data science methods within bioethics. It is well-known that definitions can serve different functions, for instance telling us what a thing is or, more tentatively, guiding the “search for an explanation of meaning” (Gupta and Mackereth 2023). Similarly, we take it that the definition of Digital Bioethics as an interdisciplinary field serves at least four functions. First, the definition might delineate boundaries of inquiry to identify tangential topics and approaches, which are more or less outside the scope of bioethicists’ interest and competence, i.e. a definition can serve to better organise knowledge. Second, it might contribute to the substantive and methodological foundations of research to include interdisciplinary competence to tackle the ethical challenges of current digital medicine and healthcare. In this sense, a definition can facilitate access to knowledge from other research fields. Besides these systematic and collaborative functions in terms of shaping a scientific community, a definition might, third, facilitate the development, translation and deployment of specialised methodologies, in order to epistemically approach digital and non-digital research topics. Fourth and finally, it might strategically help in securing institutional as well as public and political support by articulating the significance and specific character of the field, aiding its further development. In the following, we will explore the advantages and disadvantages of the systematic, collaborative, epistemic and pragmatic functions in more detail.

Fields of study in applied ethics are often defined by their objects of inquiry such as in environmental, animal or medical ethics. In addition, however, there are also branches of applied ethics which are defined by their theoretical and methodological approaches such as empirical, narrative, phenomenological or hermeneutical bioethics. There are further examples of expanding the scope of bioethics or specifying its analytical lenses such as in feminist approaches to bioethics. These attempts react to epistemological, hermeneutical and methodological shortcomings of present approaches in bioethics given present technological and societal developments (Ursin et al. 2024). Digital Bioethics, as we understand it in this article, follows these successful examples in incorporating a methodological focus in its definition.

As in good scientific practice, the choice of methods follows the research question. However, if bioethics researchers lack sufficient knowledge of new methodological toolkits, they run risk of losing sight which methodological innovations offer fruitful ways for researching specific phenomena. The existing Digital Bioethics literature already reflects, to some extent, that many questions of interest to bioethics are mediated by digital technologies such as social media (Müller et al. 2020; Lupton 2020). In fact, it is sometimes necessary to use digital methods to research digital phenomena if, for example, access to the required data is otherwise not possible or if the amount of data prohibits manual analysis within a reasonable period. In other cases, the decision to apply advanced digital methods is not imperative but a reasoned choice can be made between “conventional” bioethics methods and their computational counterparts. Discussing Digital Bioethics as an emergent field, however, helps in informing such choices and making them more explicit as researchers become more aware of their methodological options.

New research areas arise for several reasons, including important epistemic ones. New methodologies allow to access the previously unknowable, new fields of study emerge at the intersection of established ones, or influential interest groups suddenly pay attention to new research while the cumulative effect of several factors may lead to the institutionalisation of a new field. While there is some path-dependence derived from the parent disciplines, a natural process of specialisation contributes to new fields of study. Krauss argues that we hitherto underestimate the role of “methodological revolutions” including the “digital revolution” for the development of science in particular and civilisation in general, because they are the main drivers for scientific and technological knowledge production (Krauss 2023).

Pragmatic reasons for defining a scientific field are manifold. Clear definitions help to demarcate scientific fields from non-scientific or pseudo-scientific endeavours and in maintaining the integrity of a field, distinguishing it from less rigorous forms of inquiry. Avoiding definitional ambiguities and inconsistencies also helps to uphold the Mertonian norms of science and to avoid the promotion of counter-norms such as secrecy, particularism, self-interestedness, and organised dogmatism (Anderson et al. 2010). From a pragmatic perspective, defining a scientific field is also essential for setting research priorities and directions. Funding agencies and institutions rely on demarcated research fields to allocate resources effectively and to ensure that projects align with their strategic goals (Lyall et al. 2013).

Agreeing on a provisional definition of Digital Bioethics adds substantial value for researchers working within and outside the field. For digital bioethicists, a definition provides several key benefits: referring to a definition facilitates collaboration, helps to identify research questions, design studies, and interpret results. It also might lead to the development of best practices and may help researchers develop a professional identity. For researchers not working in Digital Bioethics, a definition as narrow as necessary and as wide as possible offers several advantages as well: It helps to understand the methodological peculiarities of Digital Bioethics, facilitates inter- and transdisciplinary research and collaboration, and provides a reliable reference point for citing work in Digital Bioethics, ensuring an accurate representation of the field’s contributions to novel research. At the same time, a definition for an emerging field should be flexible enough not to exclude important types of study from the early beginning. It should also be open to scrutiny and potentially needs to be readjusted as the field further develops. Regarding the definition as suggested in this article, a deliberate decision was therefore made not to restrict the methodological side (e.g., which informatics and data technologies are used) too early. We intentionally did not limit the scope of Digital Bioethics to approaches that merely augment empirical *research*. Instead, we also included efforts that apply advanced data science to other vital areas of bioethics, such as argumentative analysis or counselling, for instance in clinical ethics.

## Topics and methods in digital bioethics

The label *Digital Bioethics* is ambiguous as the term ‘digital’ can be understood in two rather different ways. In one reading, the attribute ‘digital’ is interpreted as referring to the *object* of investigation, i.e. as reference to its research topic. The field would then be defined as being concerned with research that focuses on bioethical issues raised by the use of digital technologies – such as a lack of explainability in automated disease recognition, or privacy concerns in digitised health records. However, to avoid ambiguity, we believe it is more fruitful to refer to this field as “bioethics of the digital” and reserve the term Digital Bioethics for a reading that interprets ‘digital’ as describing the *method* that is employed for the bioethical investigation (Salloch and Ursin 2023). In this case, Digital Bioethics is an interdisciplinary field concerned with research in the bioethical domain that is conducted using advanced digital methods, for instance by computationally modelling ethical decision-making in clinical settings. Adhering to such terminology is also in line with the term “Empirical Bioethics”, referring to bioethical research that is conducted with the help of empirical methods. In the more general context of digital ethics, Ranisch et al. concisely capture the two interpretations by referring to the former as ethics *of* digital technologies, and to the latter as ethics *with* digital technologies (Ranisch, Vayena, and Buchholz (in press)).

The use of large language models in bioethics can illustrate this methodological shift in a particularly clear way. Where LLMs are used not merely as writing aids but as systems that structure, simulate, or inform ethical reasoning, they draw on moral assumptions ingrained in their underlying models (Kiehne et al. 2024; Hadar-Shoval et al. 2024; Soffer et al. 2025; Jiang et al. 2025; Pfeffer et al. 2025). Recent empirical work on “virtual digital humans” further illustrates how digital entities are perceived as occupying an intermediate moral status, neither as mere tools nor as proper moral agents, generating ethical questions that arise specifically from digital mediation (Xin et al. 2025). Reflecting on such moral tendencies and biases within tools used for bioethical inquiry, notably including which ethical theories they represent and how, requires a methodological stance that is not covered by traditional empirical bioethics using methods that are focused on reflecting on human attitudes (Braun and Clarke 2019).

**Fig. 1:**
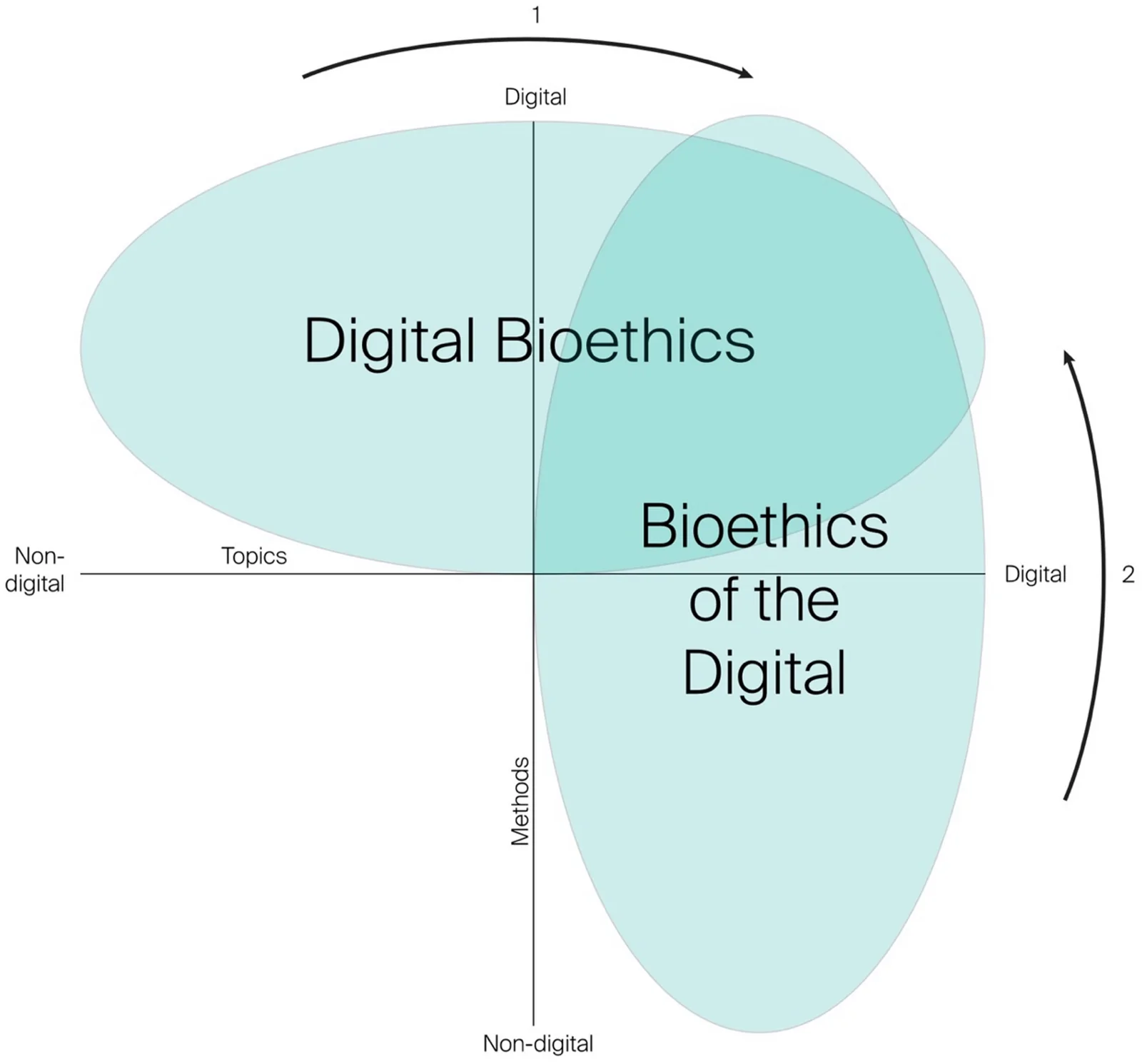
The relation of topic and method in digital bioethics

Since both topic and method can be either digital or non-digital, there are, in total, four permutations, which correspond to the four quadrants of the coordinate system below (Fig. 1, clockwise from top-left): one can employ (a) digital methods to study a non-digital topic; (b) digital methods to study a topic related to digitality; (c) non-digital methods to study a topic related to digitality and (d) non-digital methods to study a non-digital subject. According to these parameters, each bioethical research question could be positioned along the respective axes of the following coordinate system.^3^

As the figure highlights in its top two quadrants, research adopting advanced digital methods falls within the remit of Digital Bioethics, irrespective of its topic. Similarly, any research on questions touching on the digital belongs to the overarching category of bioethics of the digital, and there is an overlap between both areas, namely research performed with digital methods on topics concerning digitality. Quadrant four is left out of both categories since it does not comprise any digital elements; all bioethical research that predates the digital era would fit into this category as well as current research using conventional methods for bioethical topics that do not relate to digitality.

Importantly, the axes of the coordinate system are meant to represent the granularity of the parameters. For instance, along the topic axis, one might further distinguish topics *inherently* related to digitality such as social media communication from topics that are primarily analogue but are becoming increasingly digitised such as documentation of patient data. Such shift from one area to the other is denoted by arrow number 1. Conversely, the same kind of granularity holds true with view to methods: Some research in Digital Bioethics is merely facilitated by digital methods, for instance if LLMs are used for text production in ethical research (Porsdam Mann et al. 2023). Other research, however, is enabled by employing advanced computational methods and could not be achieved otherwise, for instance an AI-based patient preference predictor (Starke et al. 2025a). Insofar as existing research methods can move from the analogue to the digital, for instance if bioethicists use AI-supported software to code stakeholder interviews, there is also a potential shift of methods, denoted by arrow number 2.

Many advanced digital technologies such as generative AI, digital twins in health care or digital phenotyping, have only recently become widely used phenomena. As a result, new bioethical questions are emerging that differ significantly from earlier concerns. For example, issues around algorithmic bias in clinical decision support tools, the ethical use of synthetic patient data, or the implications of AI-generated medical advice raise distinct challenges not previously encountered in bioethics. They constitute topics of investigation that have not existed before and that differ in several respects from previous topics of bioethical investigation. Consequently, it appears reasonable to single out *Bioethics of the Digital* as the strand of bioethics concerned with the investigation of these topics, so as to highlight its particular focus and distinguish it from other, perhaps more traditional strands within the field.

However, the dynamic nature of the digital sphere may entail that a definition on this basis could soon become outdated: digital approaches are not only ever more prevalent but also increasingly intertwined with bioethical issues that were not digital before. At some point, distinguishing bioethical research on digital phenomena from bioethical research on non-digital phenomena may therefore be rendered impractical. Referring to *Bioethics of the Digital* would then become increasingly meaningless because it would no longer relevantly demarcate a specific strand of research. Consequently, while drawing a sensible distinction at this point in time, a definition that relies on a classification of the research subject alone can be expected to decrease in significance over time.

The other option – defining Digital Bioethics as bioethical research that employs digital *methods* – seems therefore more robust and forward-looking. As Hofmann (2025) points out, it is in fact common practice to characterise a field of study as “following specific and well-established methodological rules” (p. 2). However, Hofmann then turns this against the field of bioethics as a whole: since he fails to identify well-established methodological rules that are common to all bioethical research, he concludes that bioethics cannot be characterised as a coherent field of study. While this – if true – may be problematic for the field of bioethics, Hofmann’s remark clearly reveals the benefit of embracing this second interpretation of ‘digital’ in a definition of Digital Bioethics: by making methodological commonalities explicit, there is a convincing case to be made for demarcating the sub-field of Digital Bioethics from the larger field of bioethics based on its method.

## Learning from other fields

Bioethics, like many other fields before, is increasingly harnessing the benefits of digital and computational advances for its research. Yet, it is far from the first field to do so. In this section, we therefore explore digital approaches used in other, yet kindred, academic fields, and assess what each of them can teach us for critically assessing and further developing Digital Bioethics. We commence with Digital Humanities and experimental philosophical bioethics before turning to debates in computational medicine and digitised biology. In doing so, we capture some major fields of reference for bioethics, namely the humanities (and philosophy in particular) as well as clinical medicine and digitised healthcare. Topics that have been extensively discussed in these domains will then allow us to draw conclusions for the improvement of Digital Bioethics.

### Digital humanities

Given the significant role that philosophical reasoning and humanistic interpretation play in bioethics, it seems prudent to consider the implications of digital developments in this area from the perspective of Digital Humanities (DH). The field of DH addresses the increased availability of machine-readable data and the growing wealth of computational tools in various humanities disciplines. It is noteworthy that, in contrast to the natural and life sciences, this represents a relatively novel development, given the absence of a tradition of quantitative and empirical approaches within the humanities (Lauer 2013). However, this quantitative aspect is not the sole defining feature of DH. The prefix “digital” has been interpreted in diverse and sometimes conflicting ways. Depending on context, it has encompassed the use of digital tools for teaching and collaborative scholarship in the humanities, the creation of research software for textual or archival analyses, or the application of sophisticated machine learning models to large-scale datasets. These varying interpretations have led to ongoing debate about what exactly constitutes Digital Humanities, reflecting tensions between traditional scholarly practices and newer, computationally driven approaches (Terras et al. 2013). Frequently, these discussions culminate in a pivotal question: “Who’s in and who’s out?” (Ramsay 2013). To conclude this debate, the concept of a “big tent DH” has been proposed, which is open for everybody and as such centred on the integration of diverse digital approaches while deliberately blurring the boundaries of the field, as Terras has argued (Terras 2011). However, if DH is to be recognised as a discipline in its own right, as suggested by Luhmann and Burghardt (2022), a more detailed definition is necessary. Accordingly, Burghardt proposes a threefold categorisation of the diverse digital practices in the humanities (Burghardt 2020). These categories are:

1. “humanities of the digital”, which address the study of digital cultural phenomena, such as online communication or digital games from a humanities perspective, which might be media studies, literary studies, art history, or anything else;
2. “digitised humanities”, which deal with the creation, use, and exploration of digitised resources, including databases, digital libraries and in particular annotated collections and digital editions;
3. “numerical/computational humanities”, which approach humanities research questions through computational models and algorithmic procedures, typically using large corpora and datasets.

Transferring this framework to Digital Bioethics can be useful to elicit various digital perspectives there and can further clarify our demarcating approach suggested above. (1) In analogy with the “humanities of the digital” *bioethics of the digital* is often concerned with ethical issues surrounding digital technologies in healthcare. The use of AI in healthcare, health-related communication on social media platforms and online forums or concerns about the digital collection of health-related personal data would fall into this category. Such research might even profit from further attention towards the specific nature of digital cultural phenomena and data as opposed to conventional healthcare. (2) In a tentative approximation to the terminology of digital humanities, *digitised bioethics* may denote shifts from non-digital to digital as indicated by the arrows in Fig. 1. A potential example could be the analysis of a digitised collection of patients’ informed consent with assistance from an LLM-based chatbot, moving away from the analysis of analogue pen-and-paper consent forms. (3) Category three, finally, is in line with our proposed definition of Digital Bioethics, using different computational tools and machine learning models to analyse large amounts of data. Such an approach of *numerical/computational bioethics* heavily relies on the availability of digital data for analytical purposes but can also be utilised for data preparation, preprocessing, annotation, and classification. Computational tools may also be used in preliminary stages of systematic statistical analyses, for more exploratory purposes, as has been suggested by concepts such as scalable reading or visual analytics.

### Experimental philosophical bioethics – (Bio)XPhi

Earp and colleagues proposed a new framework for the use of empirical methods in bioethics, called experimental philosophical bioethics or bioxphi (Earp et al. 2020). Specific to the experimental approach is the contrastive vignette technique (CVT), wherein certain stimuli of a situation are systematically manipulated to identify the particular factors and processes that shape moral judgements (Reiner 2019). By using controlled experiments bioxphi aims to identify what factors influence people’s normative beliefs and decisions. For example, how do doctors determine what constitutes a harm when conflicting values are at stake, and what factors affect the weights they assign to each in terms of magnitude or importance (Earp and Shaw, 2017)? When policymakers draw on public attitudes to decide about the permissibility of using certain biomedical or neuroscientific technologies, what actually shapes the public’s moral attitudes toward these technologies (Mihailov et al. 2021a, b)? So, bioxphi inquires whether an identified influence constitutes reliable or unreliable grounds for our moral judgements.

Naturally, the experimental use of contrastive vignettes induces this species of empirical bioethics to focus on moral judgement as it responds to text-based scenarios. Pavarini and colleagues (Pavarini et al. 2021) argued that immersive digital tools can effectively recreate real-life situations, allowing users to experience them, make choices within them, and understand their complexity. They questioned the contrastive vignettes technique because hypothetical scenarios, typically presented through short written descriptions, lack the contextual grounding of everyday sensory experience, and also highlighted the practical use of their approach in the context of neuroethics (Lyreskog et al. 2023).

To meet the ecological validity challenge, bioxphi has integrated digital technologies and expanded its area of inquiry (Mihailov et al. 2021). For example, virtual reality technologies have helped us to better understand how people’s actions in specific situations influence their moral judgements, something we could not fully explore before. We can sometimes figure out which factors influence someone’s moral choices only after they have experienced the contextual richness of a situation. Digital Bioethics can recreate real-life pressures, like time constraints and uncertainty, while also allowing people to interact with an environment using sight and touch. The immersive power of digitally simulated experiences helps us discover intriguing connections between what people do in a situation and how they make moral judgements. Within moral psychology, researchers have simulated sacrificial dilemma scenarios developed by armchair philosophers (Francis et al. 2016). The emotionally arousing nature of virtual reality generated different behavioural responses from choices made using text-based methods. Given the discrepancy between moral thought and moral action, digital technologies are well suited to develop a comprehensive understanding of people’s behavioural tendencies and declarative attitudes toward bioethical questions.

The development of digital tools for empirical research has created sensory-rich environments which allow us to explore more realistic moral judgements and behaviours, for instance in augmented or virtual reality (Zahiu et al. 2023; Starke et al., 2025b). Instead of seeing digital and traditional experimental methods as competing approaches, experimental bioethics has opened up to digital technologies in the experimental design to explore how lived experience influences moral judgement and behaviour.

### Computational medicine

Digital technologies have also received ample attention in medicine itself. Already in 2000, the notion of digital health was coined to describe medical services relying on digital technologies (Mathews et al. 2019), and also in biomedical research, computational approaches to medicine have gained increasing prominence ever since (Tourassi 2023). Owing to improved computational capacities and the increased availability of biomedical data, the potential of these approaches seems wide-ranging, promising more accurate disease classifications, personalised therapies tailored to individual patients, and more accurate prognostics concerning the course of a medical condition (Giuffrè and Shung 2023). In consequence, dedicated research areas have been established across medical specialties, whether in cardiology, epidemiology, gynaecology, pathology, or oncology, to name just a few.

A particularly instructive field of comparison for our discussion of Digital Bioethics can arguably be found in computational psychiatry (Huys et al. 2016). This established field integrates mathematical models and data-driven approaches to better understand, diagnose, and treat psychiatric disorders. Among the different medical specialties, psychiatry and its entangled relation with the humanities make it particularly prone to draw lessons for bioethics, given that both deal with large amounts of text in their analyses and are characterised by fundamental conceptual disagreements. We take it that there are therefore at least two points which Digital Bioethics could learn from debates in the field.

First, computational psychiatry as a field defined by its methodological approach can to some extent remain agnostic with view to its underlying assumptions about the nature of mental disorders (Wiese et al. 2021). For instance, it can equally well draw on large datasets from neuroscience, representing a purely biological approach, or include large text corpora representing individual experiences as well as myriads of environmental factors, enabling a broader, more psychosocially minded view. In a similar way, Digital Bioethics can also accommodate various approaches to ethics and encompass diverging methodological and meta-ethical convictions. On a second, related point, computational psychiatry has sometimes been hailed as a potentially promising avenue to adjudicate fundamental conceptual disagreements in the field, for instance by allowing to identify natural kinds of mental disorders. There are good reasons to remain sceptical about such promises (Starke et al. 2023). Also in Digital Bioethics, it seems crucial to stress that fundamental conceptual disagreements will not be overcome by incorporating digital methods. Instead, as with empirical bioethics, findings from computational research are likely to inform and guide, not replace ethical deliberation (De Vries and Gordijn 2009).

### Digitised biology

Digitised biology is an interdisciplinary field that integrates digital technologies and computational methods to study, model, and manipulate biological systems. It represents the convergence of biology with computer science, engineering, and data science, encompassing a wide range of applications and activities closely related to research topics in biomedicine, including synthetic biology for designing biological systems, digital twins for simulation and prediction, genome editing technologies, and the use of digital tools in biological education and training.

We can learn valuable lessons from the transition to computational methods observed in biology about the initial scepticism and subsequent fruitful and successful integration of the new methods that can also shed light on the current meta-debates in bioethics, and specifically for some of the resistance the notion has recently experienced (De Proost and Provoost 2024). This shift parallels the debate in bioethics regarding “big” versus “small” science, a transition described by Derek de Solla Price (Solla Price 2019). In the 1960s, biology experienced a similar debate, initially marked by resistance to digital innovations being introduced into traditional scientific practices. Over time, these innovations were ultimately accepted and proved to be highly beneficial.

While offering immense potential for advancements in various fields, digitised biology also presents ethical challenges. These include ensuring informed consent from participants regarding the use of their biological data, protecting the privacy and security of sensitive genetic information, and addressing potential biases in data and data models that could lead to discriminatory outcomes. Here, Digital Bioethics can enter into a lively exchange on a wide range of topics.

Paradoxically, initial objections included concerns that computers might replace researchers altogether (Strasser 2010), a sentiment echoed today e.g. by Rahimzadeh et al. with regard to Digital Bioethics (Rahimzadeh et al. 2022). Additionally, the role of algorithms was often minimised, regarded merely as the “passive” function of a large-scale calculator (Strasser 2010).

Initially, computers in computational biology were primarily used to compile existing research data, exemplified by the groundbreaking “Atlas of Protein Sequence and Structure” published in 1965. However, the management of this data soon began to influence and reshape research methods, leading to the development of tools such as variant detection algorithms, gene annotation, and splicing analysis tools for RNA sequencing data. This technological advancement gave rise to a new field: bioinformatics. In bioinformatics, the aspects of both “big” and “small” biology remain interconnected (Ratti 2016). Similarly, if Digital Bioethics is increasingly considered a distinct research endeavour, it is likely that both empirical and computational aspects will continue to be viewed as closely intertwined.

## What needs to be avoided? Dangers and pitfalls of digital bioethics

As explained above, Digital Bioethics is dealing with topics that are non-digital by nature (but represented in digital data) as well as with topics that are closely related to phenomena of digitality. In the latter case, it assumes that our online social life is a *sui generis* digitally mediated form of living. Social dynamics that take place in online environments become separate phenomena with a specific digital form (Jones 1997). Compared to face-to-face communication, online interaction eliminates the social cues and signals that inform how we behave towards others and respond to them. Thus, social scientists are interested in exploring the unique nature of digital spaces where individuals interact, express themselves and share ideas. Because online social life is digitally mediated, we can now investigate vast amounts of data about people’s behaviour.

However, when dealing with phenomena of the digital world, Digital Bioethics should be aware that an online behaviour is not the exclusive product of human sociality. Most social media platforms (Instagram, Facebook, Twitter/X) engineer our sociality towards more connections (Van Dijck 2013). Users adapt and respond to the technological constraints, monetising strategies or business strategies of social media platforms (Jacobsen 2023). Persuasive design influences users towards sharing content with as many people as possible and encourages them to react to what others are doing. Algorithms are designed to increase engagement at the price of spreading morally inflammatory content, mis- and disinformation or other types of morally problematic content (Mihailov et al. 2023). Moreover, algorithms incentivise users to frame their online activity in terms of building personal brands and enhance their visibility (Haidt 2022). If Digital Bioethicists mistakenly perceive such algorithmically engineered social behaviours as authentic social behaviours, this might compromise the outcomes of ethical analyses that build on the respective research results.^4^

There are also other pitfalls related to the employment of advanced data science methods in bioethics, for example in data curation and analysis that proceed in a highly automated way without the researcher being able to supervise each step being made by the machine. Challenges such as automation bias – intensively discussed in ML-supported clinical medicine (Khera et al. 2023) – might play a role in bioethics as well and this highlights the need for explainable methods together with appropriate solutions for visualisation. Furthermore, a de-skilling of bioethicists could be feared through applications that more or less autonomously take over tasks that had been performed by humans before. With respect to LLM support in academic writing, for example, we could discuss whether important competencies get lost if bioethics scholars are no longer able to do their writing by themselves to the same extent as they had been in the pre-LLM era. In addition, a de-humanisation of ethics might be feared by some and could undermine perceived accountability for digital outputs and public trust in bioethics – especially in fields that are closely related to policy or to (clinical) practice (Benzinger et al. 2023). Additional points of concern relate to ethical theory and, for example, bear on the question of which ethical theories are computable at all (Sinnott-Armstrong et al. 2021) and whether an inadvertent transformation of the spectrum of ethical approaches as applied in bioethics might happen through the increasing use of digital methods.

On the other side, it has already been argued that some of the digital projects in bioethics so far lack a clear connection to normative argument as an ineluctable part of bioethics work (Salloch 2021). Whereas not each single publication in bioethics aims for answering a normative question (but might also be situated at the conceptual, methodological or purely descriptive level) at least some perspective towards moral problems together with ideas of how to solve them could be expected. Insights from comprehensive and complex data analyses do not per se provide an answer to ethical, i.e. normative questions. Instead, researchers in Digital Bioethics from the outset need to reflect on normative-empirical collaboration that clarifies in which ways the results of data analysis can be integrated in normative arguments. Many different forms of interaction are possible in principle such as describing popular knowledge, attitudes and behaviour related to ethically-fraught issues or assessing the context and consequences of moral advice giving or describing (Leget et al. 2009). A multitude of frameworks has been suggested – and partly also applied in concrete studies – on how descriptive and normative parts in bioethics research might fruitfully interact (Davies et al. 2015). Digital Bioethics might heavily profit from drawing connections to the debates on normative-empirical integration that took place in *Empirical* Bioethics for further developing its methodology as a normatively-oriented discipline. Researchers in Digital Bioethics should be encouraged to reflect on both the contribution their data analysis makes to normative argument as well as the normative pre-conditions they buy in by applying certain digital methods. Regarding the latter point, issues of “datafication” of real persons need to be critically reflected as well as a potential confinement of ethical theories that can be reasonably applied when dealing with huge amounts of certain types of data. In addition to that, there are examples of normative “reasoning” directly embedded in digital methods that should receive sufficient attention and reflection throughout the research process. They can relate to empathetic capabilities (Germani et al. 2025; Rudra et al. 2025), for example, but also to even more technical issues such as annotated argumentation, emotional prompting or the ethical fine-tuning of LLMs (Vinay et al. 2025). Regarding Empirical Bioethics, high degrees of vagueness and uncertainty have been documented among researchers when it comes to the integration of the empirical and the normative (Wangmo et al. 2024). Most likely, the same holds true for the emergent field of Digital Bioethics so that workshops, trainings and further research on normative-empirical collaboration would be highly desirable.

Finally, options to strengthen normative-empirical collaboration itself by digital technologies could principally be considered. These could include, for example, LLMs assessing data need and data availability to answer a certain normative question or identifying descriptive parts in normative arguments which are not sufficiently backed-up by empirical evidence. At this juncture, however, we think that the conceptual integration of descriptive and normative parts in bioethics mostly remains with human researchers who could potentially (and if technology further develops) be supported by computational means.

## What does digital bioethics need to flourish?

Digital Bioethics has recently gained momentum, but for the field to fully develop, key conditions must be met. To this end, the field may learn from the previous integration of social science methods into bioethics and the discussions about the development of empirical bioethics (Frith and Draper, 2017). In the following, we will first focus on improvements needed with specific view to the digital domain, regarding research infrastructure, data accessibility, and academic training, before turning to broader concerns with institutional support and methodological rigour, drawing on broader bioethics literature discussing its aims and methods (Mertz et al. 2014; Frith 2024). Such debates about the core of bioethics are hardly new, given that the field itself emerged as a distinct area of research and practice when scholars from various disciplines “migrated” and formed the field that is today known as bioethics. These disciplines typically included philosophy, theology or medicine. Over time, the field has expanded to include contributions from the social sciences, particularly with the “empirical turn”, where socio-empirical methods increasingly began to inform bioethics. Throughout this development, resistance to its practice has been a constant companion, for as the anthropologist Marilyn Strathern once noted, “one knows one is in an interdisciplinary context if there is resistance to what one is doing” (2005: 103, cit. in Frith and Draper, 2017: 236) – an insight that may seem encouraging to further pursue the endeavour of digital bioethics in reflectively dealing with criticisms.

The rise of Digital Bioethics now further reshapes this interdisciplinary foundation, requiring new academic structures, collaborations, and qualifications to equip scholars with the necessary skills to utilise digital technologies and research data (Schneider et al. 2023). A crucial part of research infrastructure that Digital Bioethics requires to advance relates to access to and availability of computational hardware, software, and data. Initiatives such as the FAIR Data Principles (Findable, Accessible, Interoperable, and Reusable) already provide a useful framework for the latter, ensuring that data can be effectively utilised in research (Wilkinson et al. 2016). As research into ethical questions using digital technologies progresses, ensuring that high-quality, accessible data is as openly available as possible with view to potentially disruptive technologies (Spitale et al. 2024) will be critical to let scholars engage in this field.

Beyond infrastructure, an important condition for Digital Bioethics’ flourishing relates to academic training. Traditionally, bioethicists have been trained across the (biomedical) sciences and humanities. However, the emergence of Digital Bioethics demands an additional layer of expertise. Scholars need proficiency not only in normative and empirical approaches but also in digital methods such as data analysis, machine learning, or computational modelling. This requires specialised training. While interdisciplinary postgraduate programs in bio- and medical ethics have been established, focusing on both empirical and normative dimensions, there remains a lack of programs that specifically address the intersection between bioethics and computational and data-rich methods. Although fields like Digital Humanities have begun offering training in data science and digital methods for humanities scholars, equivalent programs for bioethicists are still missing.

Another potential field of action in Digital Bioethics lies in the development of standards for research practice. In Empirical Bioethics, various approaches have been made to formulate appropriate research standards that account for the interdisciplinary nature of the field as an empirical-normative hybrid (Mertz et al. 2014; Ives et al. 2018). In the development of such guidance a key challenge lies in the task not to unduly narrow the field but to allow for methodological diversity while still upholding high scientific standards (Carter 2018). In this article, Digital Bioethics is used as an umbrella term, including extremely different activities such as online data analysis, AI-supported scholarly writing, or clinical ethics support. For the development of standards for research (or practice) further differentiations between these fields of application need to be made. Furthermore, profound knowledge of standards in the parent disciplines (e.g., ethics and informatics) is needed together with a deep interdisciplinary reflection on the specific situatedness of the respective activity that is about to be standardised.

In the broader field of the ethics of the digital applications in healthcare, many discussions have unfolded outside traditional ethical research institutions (Mahieu et al. 2018). To prevent such fragmentation and to ensure that Digital Bioethics is integrated into the wider bioethics landscape, formal academic collaborations and institutional support must be established. This also requires creating new academic alliances. Whereas bioethics research has traditionally been housed within medical schools or philosophy departments, there is now a growing need for collaboration with departments of computer science and engineering. These connections could provide bioethicists with the technical expertise needed to engage meaningfully with data research.

Going beyond training and collaboration, the recognition of Digital Bioethics by the wider scientific community is essential for its growth. This is not to claim that one should consider Digital Bioethics a discipline of its own – a status that is even contentious with view to empirical ethics and bioethics in general (Hofmann 2025; Frith and Draper 2017). Still, a structured reflection on the relation between digital tools and normative questions, the relevance of its research, its interdisciplinary approaches, and its underlying ethos seem conducive to its success and wider recognition, mirroring roadmaps for empirical bioethics (Mertz et al. 2014). In terms of scientific rigour, it is also crucial to not only further develop methodologies that incorporate digital data, including big data, into ethics research but to also establish quality standards such as reporting guidelines for data-driven methods. A useful comparison can be drawn with the field of xphi, which, especially in its early days, has been accused of the “careless emulation of poorly comprehended practices” (Woolfolk 2013) from other disciplines or of just being “bad psychology” (Cova et al. 2021). However, over time, it has gained credibility, offering insights into the challenges that Digital Bioethics might face as it seeks to establish itself as a rigorous field of study. Ideally, this development will move hand in hand with establishing venues for presenting and publishing research drawing on digital methods, as well as increased support from funding agencies and professional societies.

If these conditions are met, there are not only prospects for letting bioethics further develop as an academic discipline but positive effects on digitised healthcare must be expected as well. The normative goals of bioethical scholarship relate to the responsible use of AI technology serving the needs of patients, healthcare professionals and other stakeholders. Increased digital competencies on the side of bioethicists and a widened methodological spectrum will greatly contribute to adequate ethical oversight on AI technologies that touch on ethically sensitive issues in patient care. In this spirit, Digital Bioethics may also contribute to the aims of translational bioethics, i.e. a practice of bioethics that explicitly aims to bridge ethical theory and practice and to deliver relevant insights and tools for real-world application (Frith 2024). As recent work on AI-supported preference prediction in policymaking illustrates, a digitally enabled normative analysis could potentially even be integrated into real decision-making processes, for example by supporting reflective equilibrium-based policy development (Lim and Savulescu 2025). Digital Bioethics may therefore provide key advancements for ethical oversight and bioethics needs to become technologically savvy to fully meet its goals in a rapidly evolving field.

## Footnotes

1 This development is accompanied by the computational and data science communities increasingly becoming aware of ethics issues emerging from technological advances, such as ‘opaque’ deep learning networks, ‘theory-free’ Big Data approaches, or ‘ethical dimensions and biases’ of Large Language Models.

2 For recent work on the systematic analysis of public media debates in bioethics and how to methodologically integrate them into an empirically informed bioethics see also Ellerich-Groppe and Zimmermann (2025).

3 Method and topic are of course not exhaustive to classify and demarcate the large area of bioethics though. For instance, one could easily add dimensions to our graphic that represent different traditions of ethical reasoning.

4 Crucially, this is not to say that offline behaviour is inherently more authentic than online behaviour; yet, algorithmic systems can shape self-expression in ways that prohibit straightforward attributions. Shannon Vallor’s image of the “AI mirror” may serve here as an example to raise caution towards treating AI systems based on social media data as simple window into human moral attitudes (Vallor 2024). Recent work on algorithmic personalisation also demonstrates how value-laden computational processes can systematically influence apparent autonomous expressions of preference or normative judgement, calling for caution to render them the basis of ethical decision-making (Lu 2024).
